# Regulation of *Pseudomonas aeruginosa*-Mediated Neutrophil Extracellular Traps

**DOI:** 10.3389/fimmu.2019.01670

**Published:** 2019-07-18

**Authors:** Sladjana Skopelja-Gardner, Jomkuan Theprungsirikul, Kimberley A. Lewis, John H. Hammond, Kyrsten M. Carlson, Haley F. Hazlett, Amanda Nymon, Dao Nguyen, Brent L. Berwin, Deborah A. Hogan, William F. C. Rigby

**Affiliations:** ^1^Department of Microbiology and Immunology, Geisel School of Medicine at Dartmouth, Lebanon, NH, United States; ^2^Department of Microbiology and Immunology, The Research Institute of the McGill University Health Centre, Montreal, QC, Canada; ^3^Division of Rheumatology, Department of Medicine, Geisel School of Medicine at Dartmouth, Lebanon, NH, United States

**Keywords:** cystic fibrosis, *P. aeruginosa*, lasR, neutrophil extracellular traps, NADPH oxidase pathway

## Abstract

*Pseudomonas aeruginosa* is the most prevalent opportunistic pathogen in the airways of cystic fibrosis (CF) patients. The pulmonary disorder is characterized by recurrent microbial infections and an exaggerated host inflammatory immune response led primarily by influx of neutrophils. Under these conditions, chronic colonization with *P. aeruginosa* is associated with diminished pulmonary function and increased morbidity and mortality. *P. aeruginosa* has a wide array of genetic mechanisms that facilitate its persistent colonization of the airway despite extensive innate host immune responses. Loss of function mutations in the quorum sensing regulatory gene *lasR* have been shown to confer survival advantage and a more pathogenic character to *P. aeruginosa* in CF patients. However, the strategies used by LasR-deficient *P. aeruginosa* to modulate neutrophil-mediated bactericidal functions are unknown. We sought to understand the role of LasR in *P. aeruginosa*-mediated neutrophil extracellular trap (NET) formation, an important anti-microbial mechanism deployed by neutrophils, the first-line responder in the infected airway. We observe mechanistic and phenotypic differences between NETs triggered by LasR-sufficient and LasR-deficient *P. aeruginosa* strains. We uncover that LasR-deficient *P. aeruginosa* strains fail to induce robust NET formation in both human and murine neutrophils, independently of bacterial motility or LPS expression. LasR does not mediate NET release via downstream quorum sensing signaling pathways but rather via transcriptional regulation of virulence factors, including, but not restricted to, LasB elastase and LasA protease. Finally, our studies uncover the differential requirements for NADPH oxidase in NET formation triggered by different *P. aeruginosa* strains.

## Introduction

Chronic infections by *Pseudomonas aeruginosa* are highly prevalent in lung disease of cystic fibrosis (CF) patients (1). Inflammation as a result of *P. aeruginosa* infection is mediated by neutrophil infiltration into the lungs driven by other already recruited leukocytes, bacterial peptides, as well as chemoattractant molecules released from epithelial cells (2, 3). Neutropenic mice are more susceptible to *P. aeruginosa*-related mortality in a lung infection model (4), while humans deficient in neutrophil-mediated antimicrobial mechanisms are more susceptible to *P. aeruginosa* infection (5, 6). The presence of neutrophils in CF airways helps mediate bacterial killing through phagocytosis (7), neutrophil extracellular trap (NET) formation (8), as well as release of neutrophil microvesicles (9). While neutrophils act to kill *P. aeruginosa*, they also contribute to host lung damage due to the production of reactive oxygen species (ROS) and exposure to bactericidal proteins released from neutrophil azurophilic granules (10).

*P. aeruginosa* infects up to 80% of CF patients by 20 years of age (11). *P. aeruginosa* undergoes phenotypic and genotypic changes that allow it to withstand selective pressures of the CF airway, including nutrient availability, osmotic stress, polymicrobial environment, and anti-microbial host inflammatory and immune responses (12). By acquiring characteristic mutations, *P. aeruginosa* is able to modify its motility, alginate production, or susceptibility to host anti-microbial defenses in order to establish niches of chronic infection (13, 14). Analyses of bacterial isolates from chronically infected CF patients have demonstrated a high prevalence of *P. aeruginosa* with a null mutation in the transcription factor *lasR*, a major regulator of quorum sensing and a direct transcriptional activator of numerous virulence factors (15–17).

Despite diminished virulence, LasR-deficient *P. aeruginosa* strains appear to have survival advantage and a more pathogenic phenotype. The presence of LasR-deficient *P. aeruginosa* strains in clinical isolates from chronic infections is associated with deteriorating lung function (18). Absence of LasR confers growth advantage to *P. aeruginosa* under specific nutritional conditions due to increased expression of the catabolic pathway (19), as well as enhances bacterial resistance to cell lysis, oxidative stress, and antibiotic therapy (19–21). In addition, the high selective pressure for *lasR* loss of function mutants in the CF lung has been attributed to an increase in signaling of Anr-regulated pathways, which enable bacterial growth under low-oxygen conditions, as expected in biofilms (22). A recent study has shown that absence of LasR triggers a hyperinflammatory response characterized by enhanced neutrophil recruitment into the infected airway and decreased cytokine degradation, which together result in worsened lung injury (23). However, the pathoadaptability of LasR-deficient *P. aeruginosa* strains to the anti-bacterial host immune responses in the CF lung remains incompletely understood.

NET formation has been identified as a potent bacteriostatic and bactericidal mechanism by which extruded DNA strands from neutrophils trap bacteria and subsequently subject them to anti-microbial proteins released from neutrophil azurophilic granules (8, 24, 25). Some bacteria have evolved mechanisms to evade NETs as part of adaptive mutagenesis that aids them to establish chronic colonization (26, 27). These mechanisms include NET degradation by nucleases as well as suppression of NET formation via toxin-mediated inhibition of oxidative burst (28, 29). These reports altogether prompt us to investigate the role of LasR in mediating NET formation in the interaction between *P. aeruginosa* and neutrophils, the first line of defense in the infected airway.

Here, we demonstrate that LasR-deficient *P. aeruginosa* strains harbor a limited capability to trigger neutrophil DNA release. The reduced amount of NET release caused by LasR-deficient strains is independent of the downstream quorum sensing pathways, LPS production, and bacterial motility, but is dependent on the neutrophil NADPH oxidase pathway. Our findings suggest that diminished NET release is a result of decreased expression of virulence factors, *P. aeruginosa* elastase LasB and protease LasA, in the absence of LasR. Our results also indicate that *P. aeruginosa* type III secretion system (T3SS) exotoxins stimulate the formation of NETs, suggesting that more than one single factor influences the release of *P. aeruginosa* triggered NETs.

## Materials and Methods

### Growth Conditions and Bacterial Strains

All bacterial strains used in this study are listed in Table 1 and were provided by Dr. Deborah Hogan, Geisel School of Medicine (PA14 wild type, PA14 Δ*lasR*, PAO1 wild type, PAO1 Δ*lasR*, PA14 Δ*pqsR*, PA14 Δ*rhlR*, PA14 Δ*lasR*Δ*rhlR*, PA14 Δ*lasR*Δ*pqsR*), Dr. Brent Berwin, Geisel School of Medicine at Dartmouth (PA14 Δ*popB*, PA99 wild type, PA99 Δ*exoU*, PA99 Δ*pscJ*), Dr. Dao Nguyen, McGill University (PAO1 wild type, PAO1 Δ*lasB*), and Dr. Bryan Hurley, Massachusetts General Hospital (PA14 Δ*exoU*). Bacteria were cultured overnight in lysogeny broth (LB) at 37°C and sub-cultured for 3 h in LB (1:30) at 37°C. The medium was supplemented with tetracycline (10 μg/ml) as needed.

**Table 1:** *Pseudomonas aeruginosa* strains used in the study and source.

**Strain name**	**Description**	**Ref. no**.	**Source (reference)**
PA14 wt	Wild type	DH1722	Lars Dietrich (30)
PA14 Δ*lasR*	In frame deletion of *lasR*	DH164	Deborah Hogan (31)
PA14 *lasB*::TnPhoA	Phosphatase transposon inserted into *lasB*	DH106	Fred Ausubel (32)
PA14 Δ*pqsR*	In frame deletion of *pqsR*	DH1110	Deborah Hogan
PA14 Δ*pqsR* Δ*lasR*	In frame deletion of *pqsR and lasR*	DH1111	Deborah Hogan
PA14 Δ*rhlR*	In frame deletion of *rhlrR*	DH2742	Deborah Hogan
PA14 Δ*rhlR* Δ*lasR*	In frame deletion of *rhlR lasR*	DH237	Deborah Hogan (33)
PAO1 wt	Wild type	DH1856	Deborah Hogan (34)
PAO1 Δ*lasR::tet*	*lasR*::tet	DH2400	Deborah Hogan (35)
PAO1 Δ*lasB*	In frame deletion of *lasB*	–	Dao Nguyen
PA14 Δ*exoU*	In frame deletion of *exoU*	–	Bryan Hurley
PA14 Δ*popB*	In frame deletion of *popB*	–	Brent Berwin
PA99 wt	Wild type	–	Brent Berwin
PA99 Δ*exoU*	In frame deletion of *exoU*	–	Brent Berwin
PA99 Δ*pscJ*	In frame deletion of *pscJ*	–	Brent Berwin

### Neutrophil Immunofluorescence

To detect neutrophil granular proteins, human or mouse neutrophils (250,000 cells in 0.5 ml) were left to adhere onto 13 mm coverslips for 1 h and subsequently treated with *P. aeruginosa* strains for 2 h at 37°C at multiplicity of infection (MOI) 10. Samples were fixed with 4% paraformaldehyde, washed, and blocked using 5% donkey serum (Sigma Aldrich). BPI was detected using mouse anti-BPI antibody (1:200, Santa Cruz Biotechnology, Inc.) for human neutrophils or rabbit anti-mouse BPI antibody (1:200, ABclonal), followed by donkey anti-mouse Alexa Fluor 488 secondary antibody or donkey anti-rabbit Alexa Fluor 647 (1:500, Jackson ImmunoResearch). Neutrophil elastase (NE) was detected using rabbit anti-NE IgG (Abcam), followed by donkey anti-rabbit Alexa Fluor 647 secondary antibody (1:500, Jackson ImmunoResearch). Myeloperoxidase (MPO) was detected using rabbit anti-MPO IgG (Cell Signaling), followed by donkey anti-rabbit Alexa Fluor 647 secondary antibody (1:500, Jackson ImmunoResearch). Samples were mounted using ProLong Gold Antifade Mount with DAPI (ThermoFisher Scientific) and visualized with the laser point scanning confocal microscope (LSM 510 META, Zeiss), 63X.

### Quantification of Extracellular DNA Release

Healthy human neutrophils were obtained with the consent approved by the Committee for the Protection of Human Subjects of the Geisel School of Medicine at Dartmouth. Whole blood was diluted 1:1 in RPMI and centrifuged over Ficoll gradient (GE Healthcare). The erythrocyte pellet was diluted 1:1 in RPMI and the neutrophils were separated by 5% dextran sedimentation at 4°C for 1 h. Following red blood cell lysis using 1X BD Pharm Lyse (BD Bioscience), neutrophils were resuspended in Dulbecco's Modified Eagle Medium (DMEM) and plated on a 96-well black plate (Nunc) (200,000 cells/100 μl). Cells were treated with PMA (100 nM) or different MOIs of *P. aeruginosa* strains in DMEM without serum (unopsonized) for 3 h at 37°C. Amount of extracellular DNA was quantified by addition of Sytox Green (5 μM, Invitrogen) and fluorescence measurement (485/530 nm, BioTek, Synergy HT, BioTek Instruments, Inc.). Data are represented as % maximum DNA release, i.e., % fluorescence emitted following treatment of neutrophils with 0.5% Triton X-100 for 1 min.

### NADPH Oxidase and PAD4 Inhibition

Neutrophils were pre-treated with 10 μM diphenyleneiodonium chloride (DPI, Sigma Aldrich), pyrocatechol (100 μM, Sigma Aldrich), PAD inhibitors Cl-amidine (200 μM, Cayman Chemicals), GSK199 (10 μM, Cayman Chemicals), and GSK484 (10 μM, Cayman Chemicals) or DMSO control (0.01%) for 30 min at 37°C prior to treatment with PMA (100 nM) or different *P. aeruginosa* strains (MOI 10). Extracellular DNA was measured using Sytox Green as described above.

### Mouse Neutrophil *in vitro* Studies

To measure concentration of LPS produced by *P. aeruginosa* strains, PA14 wild type and PA14 Δ*lasR* were cultured overnight and centrifuged at 12,000 rpm for 5 min. Following centrifugation, the supernatants from the overnight bacterial cultures were diluted 1:10,000 in PBS and LPS content was measured by Limulus Amebocyte Lysate (LAL) Chromogenic Endotoxin Quantitation Kit (ThermoFisher Scientific, MA, USA) according to manufacturer's instructions. To investigate the role of TLR-4 in NET release, neutrophils were obtained from wild type and TLR4 knock-out B6 mice (Jackson Laboratory) as previously described (36). Briefly, naïve mice were injected intraperitoneally with 1 ml 4% thioglycollate solution and peritoneal cells were harvested 12–18 h later by peritoneal lavage. Neutrophils were resuspended in DMEM and treated with LPS (10 μg/ml, *E. coli*), PMA (100 nM), or *P. aeruginosa* strains at MOI 10 for 3 h at 37°C. *P. aeruginosa* strains tested were PAO1 wild type, PAO1 Δ*lasR*. PA14 wild type, and PA14 Δ*lasR*. Extracellular DNA release was quantified by Sytox Green as above.

### Neutrophil Treatment: *P. aeruginosa* Proteases and Exotoxins

*P. aeruginosa* PA14 Δ*lasR* strain (MOI 10) was supplemented with exogenous recombinant LasB (4 Units, EPC Elastin Products Company, INC or 0.3–6.6 nM, MybioSource INC) or exogenous recombinant LasA (1,000 nM, made in yeast and endotoxin free, MybioSource INC) (30 min at 37°C). Neutrophils were added (200,000 cells/100 μl) and treated for 3 h at 37°C. Release of NETs was measured using Sytox green as described above. The role of exotoxins in NETs induction was tested by treatment of neutrophils with *P. aeruginosa* strains (MOI 10) deficient in *exoU* (lacking exoU cytotoxin), *popB*, or *pscJ* (non-functional Type III secretion system, T3SS).

### Evaluation of Bacterial Swimming Motility

Swimming motility was evaluated as described previously (37). Briefly, a single bacterial colony was picked from an LB agar plate (1.5% agar) and inoculated into the center of a 0.3% agar LB plate (Gentrox). Plates were incubated for 24 h at 37°C and then for 24 h at room temperature. Swim zone was visualized by ChemiImager 5000 software and the diameter was measured as a representation of bacterial swimming capacity from the inoculation site.

### Bacterial Protease Activity Assay

The protease profile of selected *P. aeruginosa* strains was assessed using milk plates (Brain Heart Infusion with 1.5% agar and supplemented with 1% (w/v) skim milk) as previously described (38). Briefly, PA14 and PAO1 bacterial strains were cultured overnight in LB at 37°C and sub-cultured for 3 h in LB (1:30) at 37°C. *P. aeruginosa* cultures were washed with PBS then resuspended in DMEM. Each *P. aeruginosa* strain (2 ×10^6^ cfu) was added to 100 μl DMEM in at least 4 replicates. Following a 6 h culture at 37°C, 5 μl of this exponential culture was plated on milk plates in 3 replicates. Milk plates were incubated at 37°C overnight followed by image analysis. Casein degradation, observed as a zone of clearance, indicate protease activity. All strains were assayed on 3 separate days.

### Bacterial Quorum Sensing Assay

Selected bacterial strains were assessed for their ability to perform quorum sensing. Briefly, the same processed samples used to assess protease production were also used to visualize the production of acyl-homoserine lactones (AHLs) by *P. aeruginosa*. Assays were carried out on LB plates supplemented with 150 μg/ml X-gal dissolved in DMSO. An overnight culture of a PAO1 reporter strain that is responsive to both 3OC_12_-HSL and C_4_-HSL (AHLs) (39) was diluted to an OD_600nm_ = 0.01 and 100 μl was bead spread on the LB/X-gal plates to form a lawn of the reporter strain. As before, 5 μl of samples used in the protease assay were spotted on the reporter lawn in 3 replicates and the plates were incubated overnight at 37°C followed by image analysis. Quorum sensing is indicated by the blue color of the reporter lawn. All strains were assayed on 3 separate days.

### Statistical Analyses

Data were analyzed using GraphPad Prism 6 software (GraphPad Software, Inc., California, USA). Student's *t*-test, with Welch's correction, was applied to determine the significant difference between two data sets. One-way ANOVA, followed by Bonferroni *post-hoc*, was used to compare means between more than two data sets. Two-way ANOVA, followed by Bonferroni *post-hoc* was used to compare means between multiple groups studied under different conditions. *P* < 0.05 was considered significant.

## Results

### LasR Is Required for a Robust Induction of Neutrophil Extracellular DNA Release by *P. aeruginosa*

**Figure 1:**
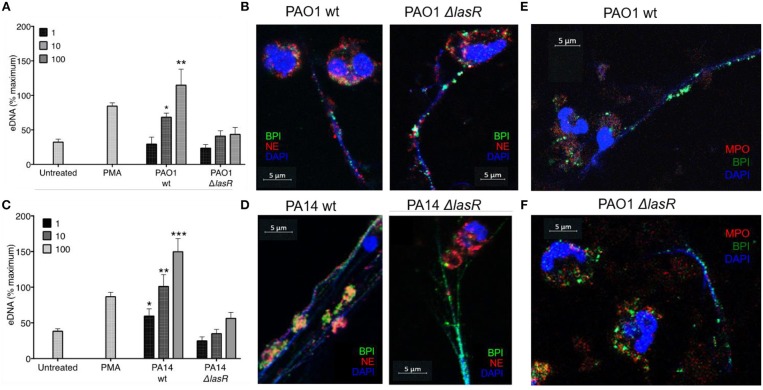
*P. aeruginosa* LasR-deficient strains induce diminished neutrophil extracellular trap (NET) release, and differential localization of BPI and NE on the extracellular DNA is strain (wild-type or Δ*lasR*) dependent. **(A)** Wild-type *P. aeruginosa* strain PAO1 (PAO1 wt) triggers greater neutrophil eDNA release than LasR-deficient PAO1 strain (PAO1 Δ*lasR*) at equivalent MOIs (*n* = 3, MOIs 1, 10, 100). **(B)** Neutrophil elastase (red, Al647) and BPI (green, Al488) localize on the NET strands stimulated by wild-type *P. aeruginosa* PAO1. However, neutrophil elastase (red, Al647) localizes mostly to neutrophil cytoplasm and membrane, in NETs stimulated by PAO1 Δ*lasR*. **(C)** Wild-type PA14 *P. aeruginosa* (PA14 wt) triggers greater neutrophil eDNA release than LasR-deficient PA14 strain (PA14 Δ*lasR*) at equivalent MOIs (*n* = 3, MOIs 1, 10, 100). **(D)** Neutrophil elastase (red, Al647) and BPI (green, Al488) localize on the NET strands stimulated by wild-type *P. aeruginosa* PA14 strain. Similarly to **(B)** neutrophil elastase (red, Al647) localizes mostly to neutrophil cytoplasm and membrane, in NETs stimulated by PA14 Δ*lasR*. **(E)** NETs induced by wild type PAO1 strain is characterized by release of BPI (green, Al488) onto the DNA strands, while myeloperoxidase (MPO, red, Al647) retains in the cytoplasm of the neutrophils. No co-localization of BPI and MPO is observed on the NETs. Nuclear DNA is stained with DAPI (blue); **(F)** PAO1 Δ*lasR* strain triggers NETs with similar phenotype to **(E)**. **(A,C)** Data were analyzed by one-way ANOVA; ^***^p < 0.0001, ^**^p < 0.001, ^*^p < 0.05; Error bars represent mean ± SEM. **(B,D–F)** Images were obtained using a 60X oil immersion objective. **(A,C)** % maximum = % of the fluorescence emitted following treatment of neutrophils with 0.5% Triton X-100 for 1 min.

In order to evaluate the role of LasR in NET formation, healthy human neutrophils were treated with wild-type (wt) or LasR-deficient (Δ*lasR*) PAO1 and PA14 *P. aeruginosa* strains. Bacteria were used in their early exponential phase (~3 h of subculture) as their ability to induce NET release is the greatest at this stage of growth (40). DNA release by neutrophils was significantly higher following treatment with the wild type strains of PAO1 and PA14 relative to their Δ*lasR* counterparts, at different multiplicities of infection (MOI 1, 10, 100) (Figures 1A,C).

By using confocal microscopy, we confirmed that the DNA release measured by the sytox assay reflects the ability of the different *P. aeruginosa* strains to trigger NETs: the DNA strands (DAPI) released from neutrophils were decorated with one or more neutrophil proteins released from azurophilic granules: neutrophil elastase (NE), BPI, and/or myeloperoxidase (MPO) (Figures 1B,D–F). Interestingly, confocal microscopy revealed differential spatial distribution of neutrophil proteins on the NETs induced by wild type vs. LasR-deficient PAO1 and PA14 strains. Following treatment with LasR-deficient *P. aeruginosa* strains PAO1 and PA14, the neutrophils not only released less DNA, but the BPI also localized on the DNA strands while NE demonstrated perinuclear staining (Figures 1B,D). In contrast, wild type strains PAO1 and PA14 triggered more DNA release with both BPI and NE localizing on the NETs (Figures 1B,D). Despite their localizing to the DNA strands, we did not observe co-localization of BPI and NE. We also observed that for NETs induced by wild-type and LasR-deficient PAO1 strains, BPI localized on the NETs while MPO staining was perinuclear (Figures 1E,F). These observations suggest that exposure to different *P. aeruginosa* strains (wild type vs. LasR-deficient) can influence both the quantity of DNA released and the localization of proteins released by neutrophil azurophilic granules. Interestingly, BPI localized to the DNA strands of NETs regardless of the stimulating bacterial strain.

### LasR Regulates *P. aeruginosa*-Mediated NET Formation Independently of Downstream Quorum Sensing (QS) Regulators: RhlR and PqsR

**Figure 2:**
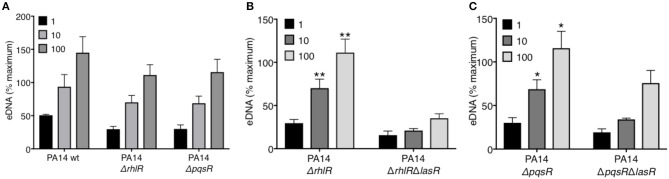
RhlR and PqsR are not required for *P. aeruginosa*-induced NET formation. **(A)** Comparable levels of NET release from neutrophils after treatment with wild-type PA14 (PA14 wt), PA14 Δ*rhlR*, or PA14 Δ*pqsR* strains at 1, 10, and 100 MOIs. **(B)** NET release is decreased in neutrophils treated with PA14 Δ*rhlR* with LasR-deficiency (Δ*rhlR*Δ*lasR*) relative to treatment with LasR-sufficient PA14 ΔrhlR bacteria, at 10 and 100 MOIs (*n* = 3). **(C)** NET release is decreased in neutrophils treated with PA14 Δ*pqsR* strain with LasR-deficiency (Δ*pqsR*Δ*lasR*) relative to treatment with LasR-sufficient PA14 Δ*pqsR*, at 10 and 100 MOIs (*n* = 3). **(A–C)** Data were analyzed by two-way ANOVA; ^**^p < 0.01, ^*^p < 0.05; Error bars represent mean ± SEM. % maximum = % of the fluorescence emitted following treatment of neutrophils with 0.5% Triton X-100 for 1 min.

LasR occupies a high regulatory position in the hierarchy of the quorum sensing network in *P. aeruginosa* and regulates the downstream QS systems: Rhl and Pqs. Using a reporter system, we confirmed the absence of quorum sensing in our LasR-deficient PA14 and PAO1 strain, which lacked the ability to produce AHL (Supplemental Figure 3). Mutations in the quorum sensing genes of *P. aeruginosa* have been implicated in its pathoadaptability (18, 20, 41). Therefore, we investigated the role of the two regulatory proteins, RhlR and PqsR, in the presence or absence of LasR, in inducing NET release. Absence of RhlR or PqsR alone from PA14 bacterial strain did not abrogate the ability of *P. aeruginosa* to mediate robust DNA release (Figure 2A). However, the absence of both LasR and the downstream QS pathways, i.e., Δ*rhlR*Δ*lasR* and Δ*pqsR*Δ*lasR*, significantly decreased the capacity of *P. aeruginosa* strain PA14 to trigger DNA release (Figures 2B–C). These findings demonstrate that the requirement for LasR in mediating NET formation is not through transcriptional activation of the downstream quorum sensing systems Rhl and Pqs.

### *P. aeruginosa* LasR Regulates NET Release Across Host Species, Independently of Bacterial Motility and LPS

A recent study has identified that bacterial motility is essential for the ability of *P. aeruginosa* to trigger NETs (40). This prompted the possibility that LasR regulates NET formation by regulating bacterial motility. To address this, a standard motility assay was performed, which demonstrated that wild-type and all mutant *P. aeruginosa* strains used in this study are motile, thus eliminating the possibility that loss of *lasR* gene inhibits NET release due to changes in bacterial swimming motility (Supplemental Figure 1).

**Figure 3:**
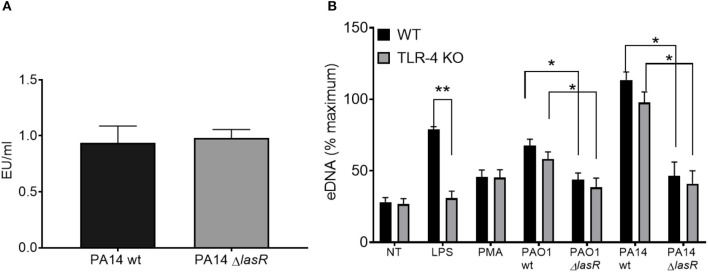
Differential NET release with *P. aeruginosa* strains occurs in mouse and human neutrophils and is LPS-independent. **(A)** Wild-type and LasR-deficient PA14 *P. aeruginosa* strains produce equivalent concentrations of LPS during overnight bacterial cultures, as measured by Limulus Amebocyte Lysate assay and analyzed by Student's *t-*test (*n* = 3). **(B)** Wild-type PAO1 and PA14 *P. aeruginosa* strains trigger greater NETs than their LasR-deficient counterparts, from both wild-type and TLR4 knock out mouse neutrophils. LPS and PMA (100 nM) were used as controls (*n* = 3). Data were analyzed by two-way ANOVA; ^**^p < 0.01, ^*^p < 0.05; Error bars represent mean ± SEM. % maximum = % of the fluorescence emitted following treatment of neutrophils with 0.5% Triton X-100 for 1 min.

Since LPS has been described as a potent NET stimulant and is potentially regulated by quorum sensing (42, 43), we investigated whether the differential effect of *P. aeruginosa* strains on NET induction was LPS dependent. First, we demonstrated that PA14 wild type and PA14 Δ*lasR* strains expressed comparable levels of LPS by Limulus Amebocyte Lysate assay (Figure 3A). Second, DNA release from wild-type (TLR-4 sufficient) and TLR-4 knockout (TLR-4 deficient) murine neutrophils was analyzed. Wild type PAO1 induced comparable levels of DNA release from both TLR-4 sufficient and -deficient mouse neutrophils, while Δ*lasR* PAO1 triggered less DNA extrusion, independently of TLR-4 expression (Figure 3B). A similar relationship was observed with PA14 strains (Figure 3B). As expected, LPS triggered DNA release from wild-type mouse neutrophils but not from TLR-4 deficient ones (Figure 3B). Immunofluorescence staining confirmed NETosis in mouse neutrophils, characterized by release of DNA strands decorated with BPI, protein stored in azurophilic granules (Supplemental Figure 2). These data demonstrate that diminished NET induction seen with LasR-deficient *P. aeruginosa* strains is LPS independent and conserved across murine and human species.

### *P. aeruginosa* LasB Elastase and LasA Protease Contribute to *P. aeruginosa*-Induced NETs

**Figure 4:**
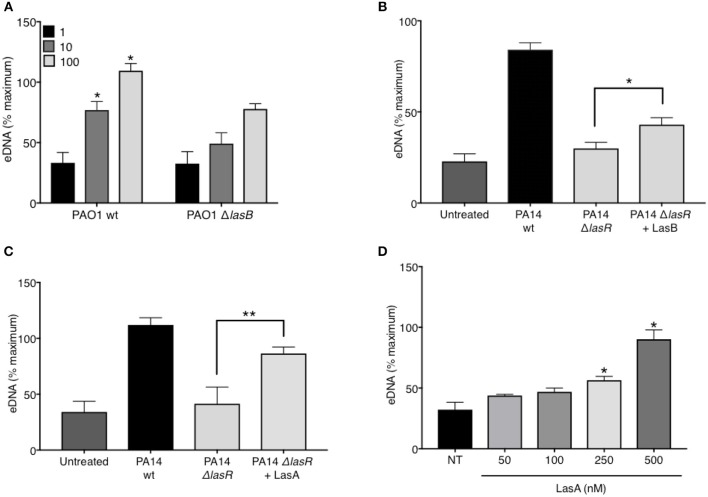
*P. aeruginosa* elastase LasB and *P. aeruginosa* protease LasA contribute to *P. aeruginosa*-induced NET formation. **(A)** Wild-type PAO1 *P. aeruginosa* (PAO1 wt) triggers greater NET release from neutrophils than LasB-deficient PAO1 strain (PAO1 Δ*lasB)* (*n* = 3, MOIs 1, 10, 100) as analyzed by two-way ANOVA, ^*^p < 0.05. **(B)** Addition of exogenous LasB (2 units) to PA14 Δ*lasR* strain (MOI 10) only modestly increases PA14 Δ*lasR*-induced NETs from healthy neutrophils (*n* = 3), as analyzed by one-way ANOVA, ^*^p < 0.05. Error bars represent mean ± 2SD. **(C)** Addition of exogenous recombinant LasA (500 nM) to PA14 Δ*lasR* (MOI 10) significantly increases PA14 Δ*lasR*-induced NETs from healthy neutrophils, as determined by One-way ANOVA (*n* = 3, ^*^p < 0.05, ^**^p < 0.001). **(D)** Recombinant endotoxin-free LasA induces NETs from healthy neutrophils in a concentration-dependent manner (50–500 nM), as determined by One-way ANOVA (*n* = 3, ^**^p < 0.01, ^*^p < 0.05). Error bars represent mean ± SEM. % maximum = % of the fluorescence emitted following treatment of neutrophils with 0.5% Triton X-100 for 1 min.

In addition to regulating the downstream quorum sensing elements, LasR directly controls transcriptional activation of a wide array of *P. aeruginosa* virulence factors (44). Indeed, we confirmed that our LasR-deficient PA14 and PAO1 strains lacked the ability to make proteases, demonstrating no casein degradation in the protease activity assay (Supplemental Figure 3). Since we have demonstrated that LasR does not influence neutrophil DNA release through the quorum sensing network, Rhl and Pqs, we investigated the role of LasR-controlled proteases, LasB elastase and LasA protease, on NET induction. LasB has previously been implicated in modulating the pro-inflammatory environment of the CF lung and in mediating cleavage of neutrophil-derived proteins (45). Treatment of neutrophils with PAO1 strain deficient in LasB (Δ*lasB*), at different MOIs, led to a reduced DNA release as compared to that triggered by wild type PAO1 (Figure 4A). The contribution of LasB to PA14-mediated DNA release was further evaluated by addition of exogenous LasB to PA14 Δ*lasR*. The presence of exogenous LasB allowed for a modest but significant increase in PA14 Δ*lasR*-mediated DNA release (~20%) (Figure 4B). A slightly greater eDNA release was observed using a different recombinant LasB, which demonstrated peak 40% eDNA increase, an effect that plateaued at ~3 nM (not shown). Therefore, these data suggest that LasB contributes to NET induction but, when added exogenously, is not sufficient to restore DNA release by LasR-deficient *P. aeruginosa* to the level of its wild-type counterpart. We further explored the ability of LasA protease, another virulence factor under LasR control (46), to restore the ability of LasR-deficient *P. aeruginosa* to trigger NETs. As seen with LasB, addition of recombinant endotoxin-free LasA restored the ability of PA14 Δ*lasR* bacteria *P. aeruginosa* strain to trigger DNA release, however not to the full extent of the wild-type *P. aeruginosa* strain (Figure 4C). Recombinant LasA triggered DNA release from neutrophils in a concentration dependent manner (Figure 4D).

**Figure 5:**
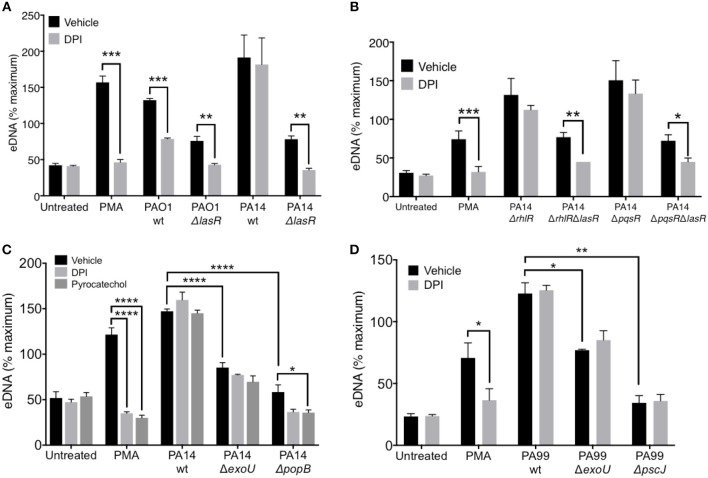
LasR but not exotoxins regulates the requirement for NADPH oxidase in *P. aeruginosa* induced NETs. **(A)** Inhibition of NADPH oxidase by pre-treatment of neutrophils with DPI (10 μM, 30 min at 37°C) decreases NET release from neutrophils treated with PMA, wild-type PAO1, PAO1 Δ*lasR*, and PA14 Δ*lasR* but not with wild-type PA14 strain (10 MOI), relative to vehicle control (DMSO) (*n* = 3). **(B)** Inhibition of NADPH oxidase with DPI (10 μM) decreased NET release from neutrophils treated with PMA, PA14 Δ*rhlR*Δ*lasR*, and PA14 Δ*pqsR*Δ*lasR* but not following treatment with PA14 Δ*rhlR* and PA14 Δ*pqsR* strains (10 MOI), relative to vehicle control (DMSO). **(C)** *P. aeruginosa* PA14 strain deficient in type III secretion system (T3SS) Exotoxin U (Δ*ExoU*), as well as strain deficient in T3SS PopB (Δ*popB*) translocon trigger less neutrophil DNA release than their PA14 wild-type counterpart. The eDNA release is not inhibitable by neutrophil treatment with DPI (10 μM) or pyrocatechol (100 μM), with an exception of PA14 Δ*popB* which appears to be inhibited by pyrocatechol. **(D)** Neutrophil DNA release triggered by wild-type *P. aeruginosa* strain PA99, as well as PA99 strains deficient in ExoU or PscJ (Δ*pscJ)* is not inhibited with DPI (10 μM). Data were analyzed by one-way ANOVA **(A,B,D)** or two-way ANOVA **(C)**; ^****^p < 0.0001, ^***^p < 0.001, ^**^p < 0.01, ^*^p < 0.05; Error bars represent mean ± SEM. % maximum = % of the fluorescence emitted following treatment of neutrophils with 0.5% Triton X-100 for 1 min.

These and other findings suggest that multiple *P. aeruginosa* components have the ability to trigger NET release and that these components might be under LasR transcriptional control. As we observed in Figures 1A,C, and 5A, PAO1 induced less neutrophil DNA release than PA14, both in the wild-type and the Δ*lasR* strains, confirming previously reported findings that cytotoxic PA14 strain is a much more potent stimulant of NETs than the invasive PAO1 strain (47). Because PAO1 is deficient in *exoU*, a type III secretion system (T3SS) effector protein (31), the diminished ability of PAO1 to stimulate NET release, relative to the highly virulent PA14 strain, prompted the possibility that T3SS exotoxins may contribute to *P. aeruginosa-* mediated NET formation (discussed below).

### LasR, but Not Exotoxins, Regulates the Requirement for NADPH Oxidase in *P. aeruginosa—*Induced NETs

While it is known that PMA induces DNA release in an NADPH-oxidase dependent manner, recent studies have suggested that the requirement for NADPH oxidase in NET formation is dependent on the stimulus (48). Therefore, we evaluated the ability of PAO1 and PA14 wild-type and Δ*lasR* mutant *P. aeruginosa* strains to trigger DNA release from neutrophils pre-treated with a NADPH oxidase specific inhibitor, diphenyleneiodonium chloride (DPI). DPI decreased DNA release triggered by PAO1 wild type and the corresponding Δ*lasR* mutant (Figure 5A). Interestingly, the effect of DPI on neutrophil DNA release triggered by PA14 was present only in the PA14 Δ*lasR* mutant strain, but not PA14 wild-type strain (Figure 5A). These data highlight the differences in mechanisms employed by PAO1 vs. PA14 in mediating NET formation but also suggest that PA14 Δ*lasR* strain acquires a PAO1-like phenotype that requires NADPH oxidase in order to trigger NETs. The ability of the Δ*lasR* mutation to confer DPI sensitivity (NADPH oxidase dependence) to DNA release from PA14 treated neutrophils was further evaluated in the context of downstream quorum sensing regulators using single RhlR (PA14 Δ*rhlR*) or PqsR (PA14 Δ*pqsR*) as well as double mutants (PA14 Δ*rhlR*Δ*lasR* and Δ*pqsrR*Δ*lasR)*. As seen with the wild-type PA14, the ability of DPI to inhibit DNA release segregated with the Δ*lasR* mutation in all the strains (Figure 5B). Furthermore, we confirmed that wild-type PA14 triggers NETs via a ROS-independent mechanism since both DPI and the ROS scavenger pyrocatechol lacked the effect on DNA release that was seen with PAO1 (43, 49) (Figure 5C). Since citrullinated histones have been found in *P. aeruginosa* induced NETs (40, 50), we examined whether PAD activity is necessary for this process. None of the *P. aeruginosa* strains required PAD activity to trigger NETs (51, 52), as indicated by the lack of effect of three different PAD inhibitors (Supplemental Figure 4).

Since the presence of intact LasR associated with ROS-independent NET formation in the PA14 but not the less virulent PAO1 strain, we examined the contribution of PA14 exotoxins. First, we demonstrated the importance of PA14 T3SS in triggering DNA release from neutrophils: PA14 deficient in the T3SS translocator protein PopB were unable to trigger robust neutrophil DNA release (Supplemental Figure 5). Moreover, the DNA release triggered by PA14 Δ*popB* was sensitive to both DPI and pyrocatechol (Figure 5C). In contrast, while the exonuclease U–deficient strain (PA14 Δ*exoU*) also induced lower levels of neutrophil DNA release relative to PA14 wild type, it was insensitive to DPI and pyrocatechol (Figure 5C). DPI was unable to prevent NET release from neutrophils treated with two other *P. aeruginosa* strains with altered T3SS: (i) PA99 ExoU-deficient strain and (ii) PA99 strain lacking PscJ, a lipoprotein component of the basal substructure of the needle complex of T3SS (53) (Figure 5D). Together, these findings provide two mechanistic insights: (i) intact T3SS is necessary for robust NET release and (ii) the requirement for ROS in *P. aeruginosa*-mediated NET release is strain-specific.

## Discussion

**Figure 6:**
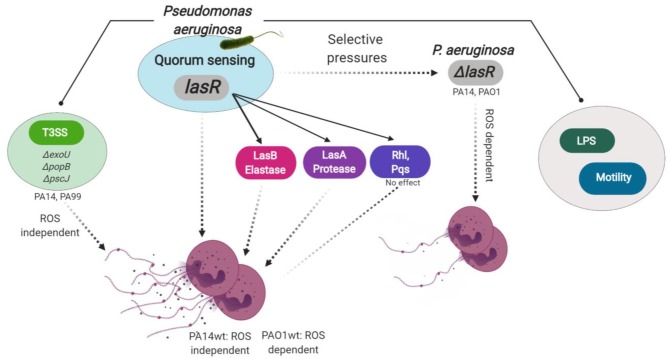
Model for *P. aeruginosa* regulation of NETosis. Master quorum sensing (QS) regulator LasR is required for robust neutrophil eDNA release by *P. aeruginosa*. LasR regulates NET formation through virulence factors: elastase LasB and protease LasA but not through the downstream QS pathways Pqs and Rhl. Under host selective pressures, *P. aeruginosa* loses functional *lasR*, which absence leads to diminished neutrophil eDNA release, independent of LPS or bacterial motility. Requirement for reactive oxygen species (ROS) in *P. aeruginosa* triggered NETosis is strain dependent. LasR sufficient (wt, wild type) PA14, but not PAO1, strain mediates eDNA release independently of ROS. Both PA14 and PAO1 LasR deficient (Δ*lasR)* strains trigger some eDNA release through a ROS-dependent mechanism. Exotoxins released through the type 3 secretion system (T3SS) of PA14 and PA99 strains contribute to the robust eDNA release independent of ROS. Figure created with BioRender.

In this study, we uncover the importance of LasR in *P. aeruginosa*-stimulated NET release. We show that NET formation stimulated by LasR-deficient *P. aeruginosa* strains appears quantitatively and qualitatively different from that seen with the wild-type strains: (i) LasR-deficient *P. aeruginosa* trigger lower levels of neutrophil DNA release and (ii) LasR-deficient but not wild-type PA14 requires NADPH oxidase for NET release. We determine that LasR regulates NET formation via mechanisms other than the downstream quorum sensing signaling systems: Rhl and Pqs. Rather, LasR appears to regulate NET release, at least in part, via virulence factors directly controlled by LasR: *P. aeruginosa* elastase LasB and *P. aeruginosa* protease LasA. Diminished NET induction by LasR-deficient *P. aeruginosa* strains is not due to loss in bacterial motility or LPS production, and is conserved across human and murine species. Thus, our findings uncover a novel role for *P. aeruginosa* LasR in regulating neutrophil function (Figure 6) and could have important implications in how this pathogen adapts to and evades innate immune defenses.

*P. aeruginosa* isolates containing inactivating mutations in *lasR* have been reported in up to 63% of chronically infected CF patients (54). Despite the loss of virulence factors resulting from dysfunctional LasR, *lasR* mutant clinical isolates in the CF airway are associated with worse lung function in both adult and pediatric CF patients (18). This paradoxical relationship suggests that the selective pressures in the CF lung favor loss of virulence factors, for which *P. aeruginosa* compensates by other pathogenic mechanisms. The pathogenicity of LasR-deficient *P. aeruginosa* has been reported in other inflammatory settings, such as severe corneal ulcer disease (55). The emergence of LasR-deficient, i.e., quorum sensing deficient, *P. aeruginosa* clinical isolates has been described as a means of “social cheating,” which *P. aeruginosa* employ to decrease their metabolic burden and gain growth advantage (56). Additionally, our findings propose a model by which loss of a functional LasR enables *P. aeruginosa* to avoid neutrophil-mediated bactericidal functions, e.g., NET release. Loss of flagellar motility has previously been shown to abrogate the ability of *P. aeruginosa* to trigger NETs (40). Since we demonstrated that LasR-deficient *P. aeruginosa* have intact swimming motility, loss of LasR provides yet another independent mechanism by which *P. aeruginosa* block NET formation. Future studies should aim to understand the survival advantages that this novel mechanism confers as well as take into account different clinical strains harboring *lasR* mutations as these vary in LasR function and bacterial compensatory mechanisms associated with different types of *lasR* mutations (54). From the perspective of host-pathogen interactions, our findings in *P. aeruginosa* reinforce the notion that mechanisms to resist NET formation are important for pathogenicity, as has been recognized in other microorganisms (57–60).

LasR is the global regulator of virulence factors that have been implicated in immune system evasion, such as elastase (LasB), protease (LasA), exotoxin A, alkaline protease (apr), and pyocyanin (61). Here, we demonstrate that LasR regulation of NET release is not through the downstream QS systems, Rhl, and Pqs, but via transcriptionally regulated virulence factors, including LasB and LasA. However, addition of exogenous LasB or LasA to LasR-deficient *P. aeruginosa* was not sufficient to fully restore the robust NET release seen with wild type *P. aeruginosa*. The presence of both LasB and LasA is most likely needed for *P. aeruginosa* stimulated NET release as studies have shown that activation of LasB is required for LasA processing and function (62). Overall, these findings suggest that multiple virulence factors, transcriptionally regulated by LasR, can play a role in NET formation. Besides LasR-controlled virulence factors, we demonstrate that the exotoxins transferred via T3SS are capable of triggering NETs. This was particularly evident in the more virulent PA14 strain, where absence of a functional T3SS led to diminished neutrophil DNA release. Much like *P. aeruginosa* mediated NET release appears to be a joint effort of multiple LasR-controlled virulence factors, multiple exotoxins are likely involved in triggering NETs as absence of ExoU alone was not sufficient to fully abrogate DNA release. While these data clearly demonstrate the ability of T3SS to trigger NETs, little is known about LasR control of these exotoxins. In fact, a recent study by Gifford et al. shows that the loss of LasR does not attenuate T3SS expression (63). Therefore, our findings point to two independent mechanisms of NET stimulation: one under the control of LasR and the other driven by T3SS exotoxins. Why the LasR-deficient strains almost entirely lose the ability to trigger NET release, despite T3SS presence, points to another complex relationship that warrants further investigation.

In addition to showing that the different genetic variants of *P. aeruginosa* influence its ability to trigger NETs, we find that they also shape the mechanisms involved in NET formation, i.e., the requirement for NADPH oxidase generated ROS. While NET formation has canonically been characterized by the release of ROS via NADPH oxidase-dependent mechanisms, new studies have shown that requirement for NADPH oxidase and ROS in NET release is dependent on the stimulus (48). Our findings extend this notion to the different strains of the same bacterial species as we demonstrate that NADPH oxidase is required for NET stimulation by PAO1 wild-type strain, a laboratory *P. aeruginosa* strain originally isolated from an infected burn wound. However, contrary to previous reports (50), PA14 wild-type strain, a more virulent *P. aeruginosa* isolate, triggered NETs via a ROS-independent mechanism in our studies. The same group also reported the inability of the PA14 strain to induce NETs in NAPDH-oxidase deficient neutrophils from chronic granulomatous disease (CGD) patients (50). Our findings raise the question of whether CGD neutrophils carry other abnormalities related to mechanisms of NETosis. In contrast to PAO1, the PA14 strain contains pathogenicity islands of genetic information that are horizontally transferred into the genome and influence pathogenicity via a broad array of highly cytotoxic exotoxins (31). Neither NADPH oxidase inhibition nor ROS scavenging inhibited DNA release stimulated by PA14 or PA99 bacteria deficient in exotoxins. In contrast, loss of LasR, but not its downstream QS pathways Rhl and Pqs, not only attenuated PA14-induced NETs but did so in an NADPH oxidase dependent manner. We conclude that one or more LasR-regulated genes, outside of the QS network, determine the extent and mechanism of *P. aeruginosa*-triggered NETs. These findings provide additional evidence that not only specific stimuli influence the mechanism of NET formation [Table 2, (48, 64)] but that different genetic variants of the same bacterial species differentially influence neutrophil functional mechanisms. This strain-dependent influence did not extend to citrullination, as treatment with none of the three PAD inhibitors inhibited eDNA release from wild-type or LasR-deficient *P. aeruginosa*. While citrullinated histones have been detected in *P. aeruginosa* triggered NETs (40, 50), lack of PAD inhibition in our studies suggests that citrullination is not required for *P. aeruginosa*-mediated NETosis. Our findings with *P. aeruginosa* parallel that published by Kenny et al. (65), who showed that citrullination does occur during NETosis triggered by numerous different stimuli but is not essential for eDNA release.

**Table 2:** Summary of NET formation and ROS requirement with exposure to different *P. aeruginosa* strains.

**HEALTHY PMNS**	**Stimulants (PA)**	**NETs release**	**NADPH oxidase dependency**
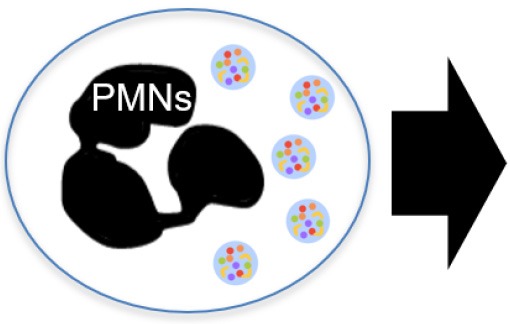	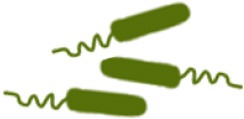	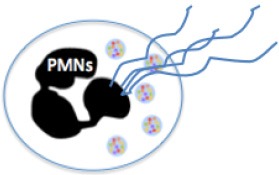	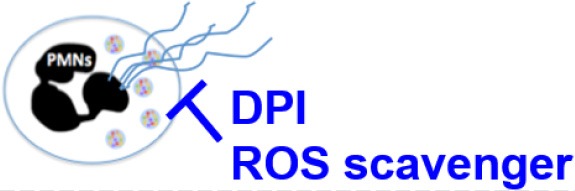
	Wild type	+++	PAO1 + PA14 -
	LasR-deficient	+	+
	RhiR/PqsR-deficient	+++	-
	RhiR/PqsR and LasR-deficient	+	+
	ExoU-deficient	++	-
	Non-functional T3SS	+	-

Despite mechanistic differences, we found that *P. aeruginosa–*stimulated NETs preferentially released neutrophil protein BPI onto the DNA strands, independent of the *P. aeruginosa* strain. This finding is interesting for two reasons: (i) BPI is a bactericidal protein that specifically targets gram-negative bacteria such as *P. aeruginosa* and (ii) presence of anti-BPI autoantibodies strongly correlates with *P. aeruginosa* infection in CF patients (66, 67). On the contrary, we observed that neutrophil elastase and myeloperoxidase are not always present on the DNA strands of *P. aeruginosa*–triggered NETs. Interestingly, autoantibodies to these bactericidal proteins are not found in CF patients (64, 66). The highly immunogenic nature of NETs (68, 69) together with the presence of a major bactericidal protein along the NET strands, provide a model by which NETs serve as an autoimmune platform in the *P. aeruginosa* infected CF airway, leading to the breaking of tolerance to BPI.

In conclusion, our study brings into the forefront a novel role for LasR in the host-pathogen interactions between *P. aeruginosa* and neutrophils. This relationship has only been examined by LaFayette et al. (23) whose study showed that LasR-deficient *P. aeruginosa* causes a hyperinflammatory neutrophil response characterized by increased cytokine production. Their findings add to the model that pathoadaptability of *P. aeruginosa* during the course of a chronic infection results in immune mediated host damage and concurrent evasion of immune defenses. While resistance to NETs or NET-mediated killing has been reported in *P. aeruginosa* clinical isolates, no specific mutation has been attributed to this mechanism of adaptability (70). We demonstrate that LasR-deficiency directly modulates NET formation, via regulation of one or more virulence factors. Finally, this work provides evidence that mechanisms engaged in NET formation vary depending on the stimulus, and can even differ between different *P. aeruginosa* variants with the same genetic background.

## Data Availability

The raw data supporting the conclusions of this manuscript will be made available by the authors, without undue reservation, to any qualified researcher.

## Ethics Statement

This study was carried out in accordance with the recommendations of Committee for the Protection of Human Subjects of the Geisel School of Medicine at Dartmouth; with written informed consent from all subjects. All subjects gave written informed consent in accordance with the Declaration of Helsinki. The protocol was approved by the Committee for the Protection of Human Subjects of the Geisel School of Medicine at Dartmouth.

## Author Contributions

SS-G, JT, and WR designed the research studies, conceptualized, and wrote the manuscript. JH, DN, DH, and BB generated the bacterial strains used in the study. SS-G, JT, KC, KL, and BB conducted the experiments. HH and AN provided processed blood samples for the study. SS-G, JT, KC, and KL acquired and analyzed the data. All authors critically reviewed the manuscript.

### Conflict of Interest Statement

The authors declare that the research was conducted in the absence of any commercial or financial relationships that could be construed as a potential conflict of interest.
