# Supplementary material for: Regulation of Pseudomonas aeruginosa-Mediated Neutrophil Extracellular Traps
Source: Front Immunol. 2019 Jul 18;10:1670. doi: 10.3389/fimmu.2019.01670 (PMC6657737; doi:10.3389/fimmu.2019.01670)
Supplement: Supplementary file 1 [file Data_Sheet_1.pdf]

Supplemental Figures

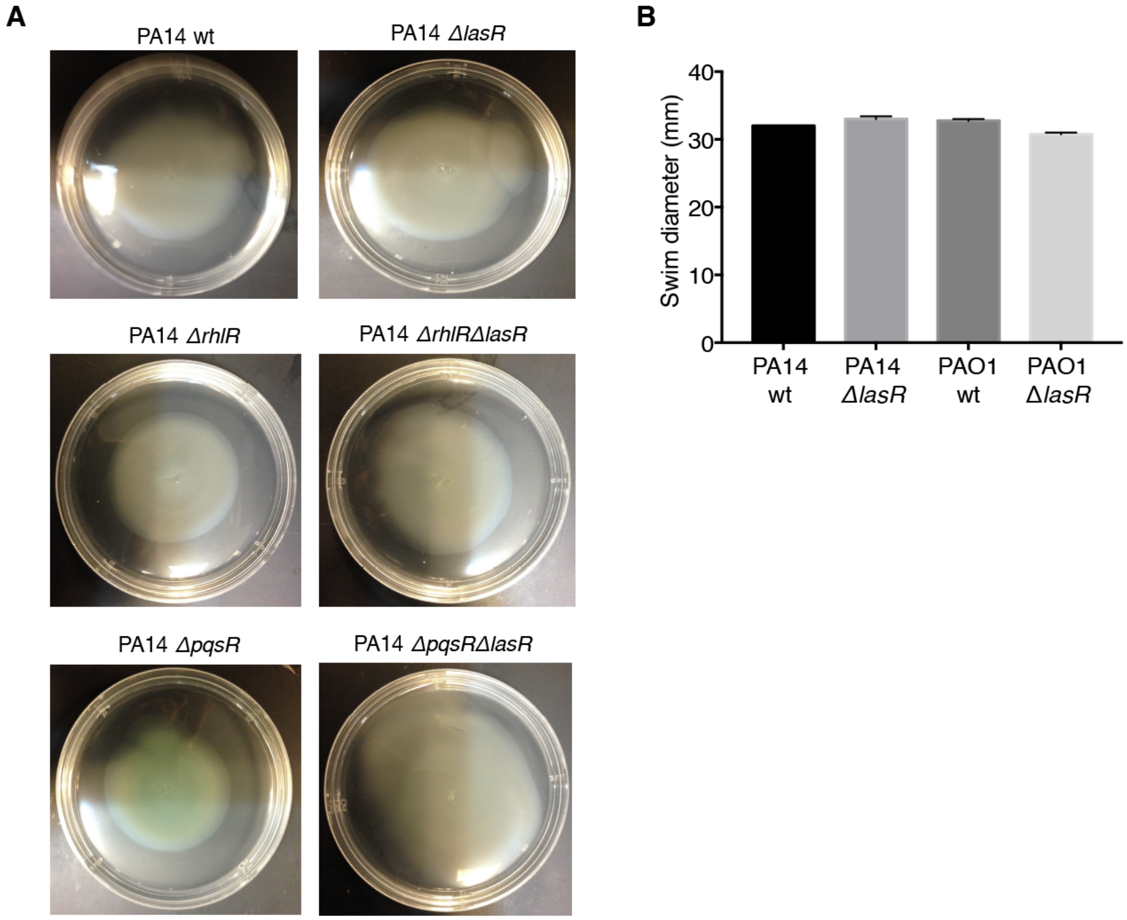

Supplemental Figure 1: LasR-deficient *P. aeruginosa* strains are motile. (A) Representative images of motility assays showing comparable motility capacities of wild-type and different mutant PA14 strains. **(B)** Quantification of swimming motility for wild-type and LasR-deficient PAO1 and PA14 *P. aeruginosa* strains (n=4). Data were analyzed by one-way ANOVA; error bars represent mean ± SEM.

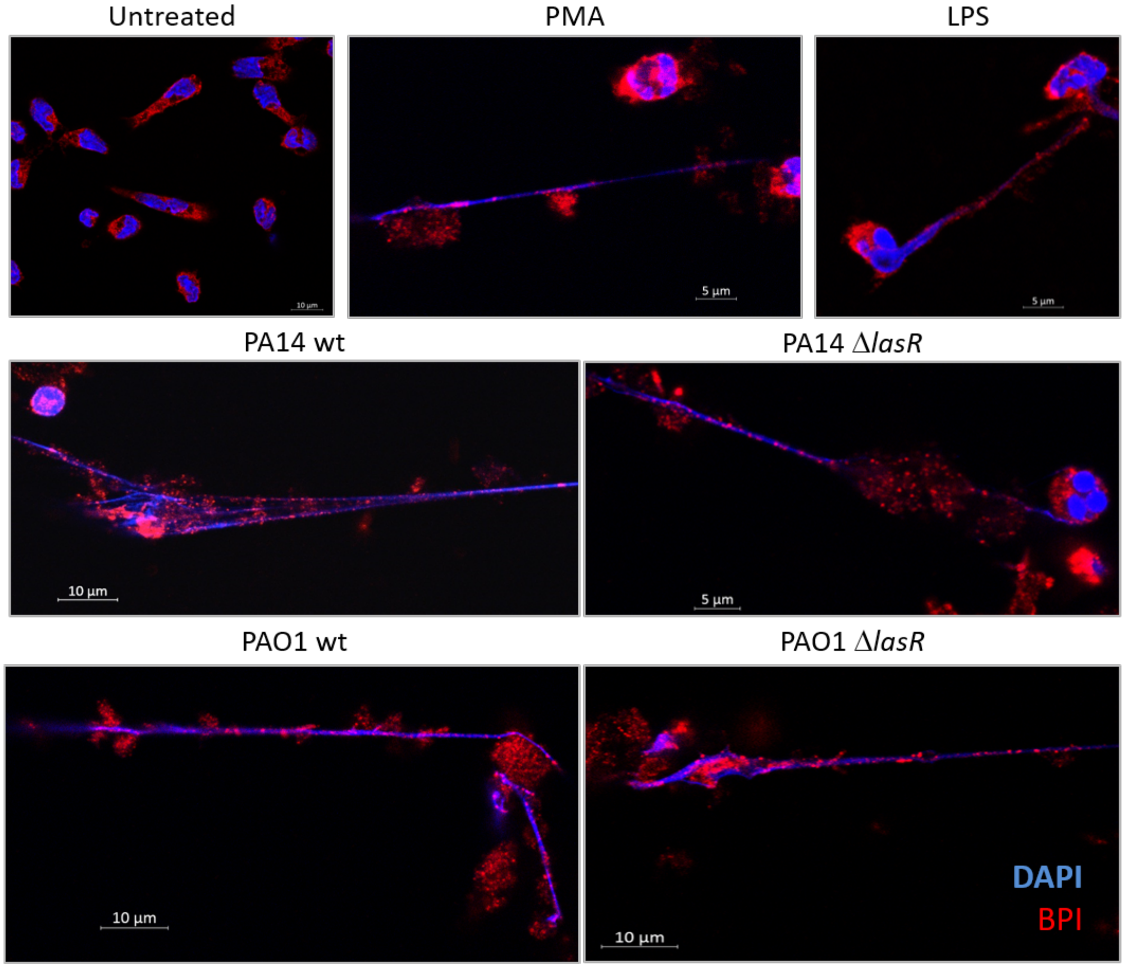

Supplemental Figure 2: *P. aeruginosa* strains trigger NET release from mouse neutrophils. BPI (red) is released onto the DNA (blue) strands from mouse neutrophils treated with PMA (100 nM), LPS (10 µg/ml), as well as PA14 and PAO1 wild-type (wt) and LasR-deficient (Δ*lasR)* and MOI = 10 for 3 hr.

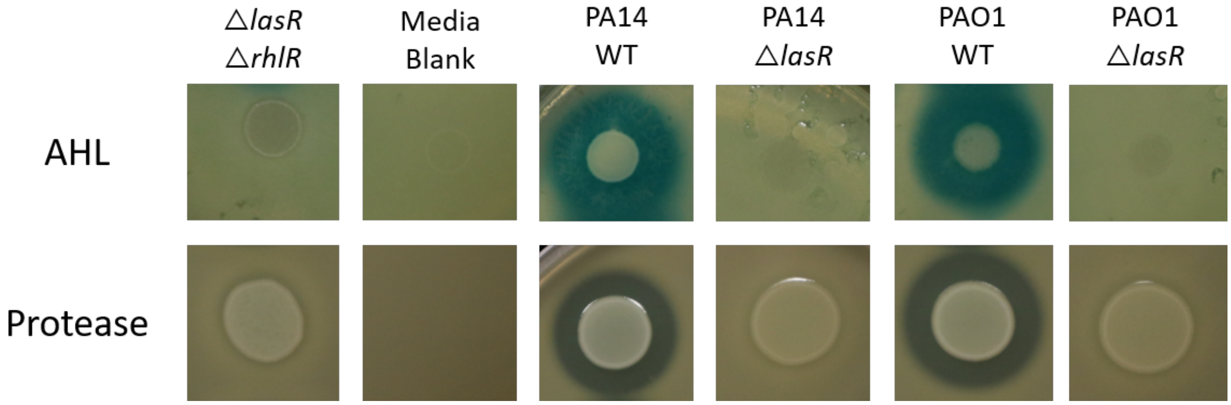

Supplemental Figure 3: LasR-deficient *P. aeruginosa* strains lack quorum sensing ability and do not secrete proteases. Only wild-type (wt) and not LasR-deficient (Δ*lasR*) PA14 and PAO1 strains produce acyl-homoserine lactones (AHL) thus activating the AHL-responsive PAO1 reporter strain (blue color, **top row**). Casein degradation, i.e. zone of clearance, was detected only in milk plates with wt PA14 and PAO1 bacterial cultures but not the LasR-deficient (Δ*lasR*) mutants, indicating lack of proteases in PA14Δ*lasR* and PAO1Δ*lasR* strains (**bottom row**).

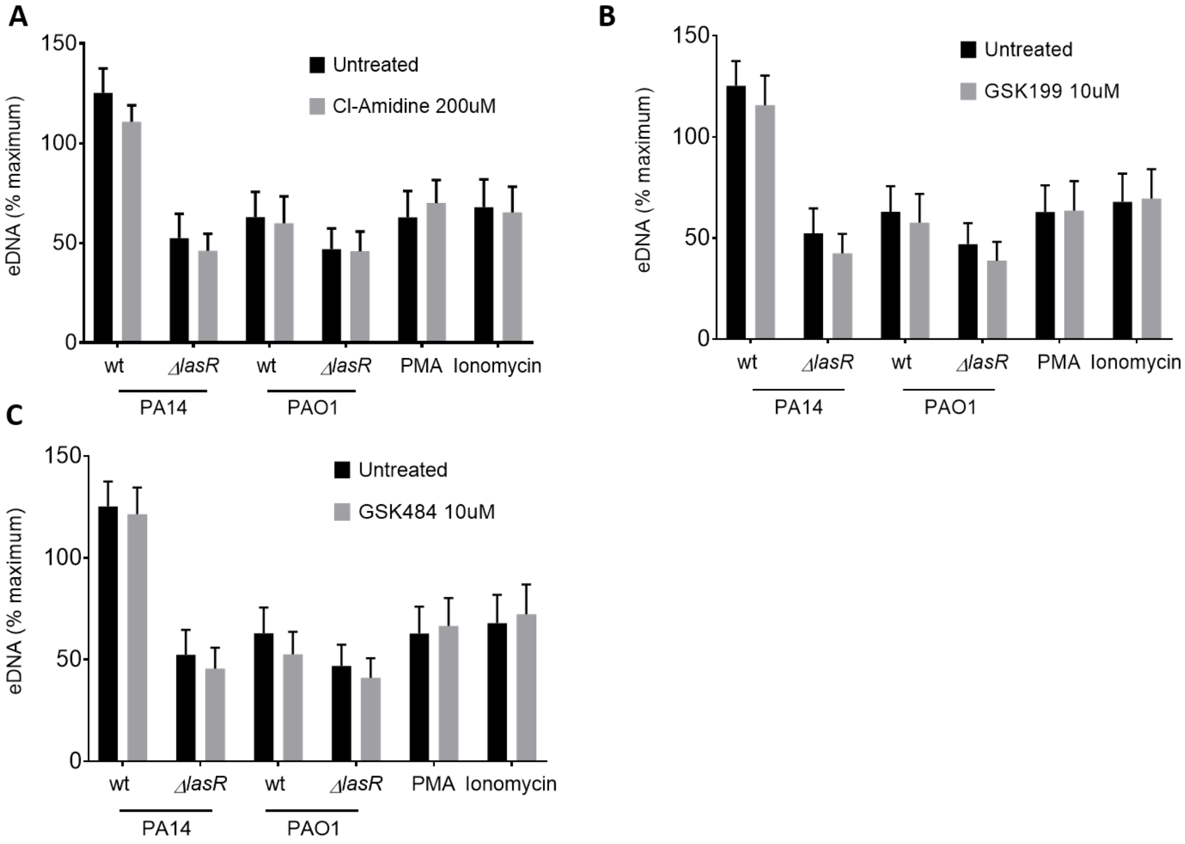

Supplemental Figure 4: Induction of NETs by PMA and all *P. aeruginosa* strains was independent of PAD enzyme activity. Inhibition of PAD enzyme activity by pre-treatment of neutrophils with (**A**) Cl-amidine (200 µM) , (**B**) GSK199 (10 µM), and (**C**) GSK484 (10 µM) does not affect the amount of NET formation when stimulated with *P. aeruginosa* wild type (PA14 wt) (MOI 10), PA14 ∆*lasR* (MOI 10), PMA (100 nM), and ionomycin (5 µM). Data were analyzed by one-way ANOVA; Error bars represent mean ± SEM.

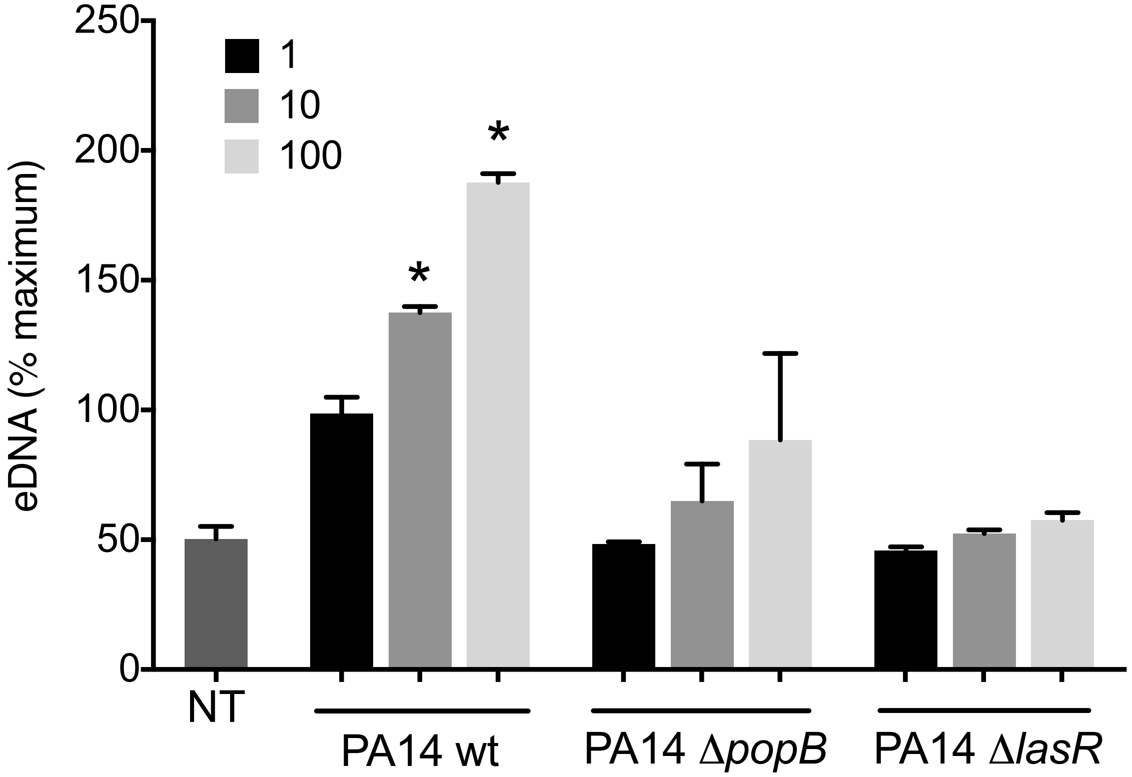

Supplemental Figure 5: PA14 Δ*popB* and PA14 Δ*lasR* induce less NET release from human neutrophils. Neutrophils from healthy donor were treated with different MOIs (1,10,100) of *P. aeruginosa* PA14 wild type (PA14 wt), PopB-deficient (Δ*popB*), LasR-deficient (Δ*lasR*) followed by incubation to form NETs. The amount of NET formation induced by both PA14 Δ*popB* as well as PA14 Δ*lasR* are less than that of their wild-type counterpart. Data were analyzed by two-way ANOVA; ***$p < 0.0001$, **$p < 0.001$, *$p < 0.05$; Error bars represent mean ± SEM.
